# Characteristics of psychotic disorders occurring during immigration: Comparative study

**DOI:** 10.1192/j.eurpsy.2021.1086

**Published:** 2021-08-13

**Authors:** I. Lajmi, S. Omri, M. Maalej Bouali, R. Feki, N. Smaoui, J. Ben Thabet, L. Zouari, N. Charfi, M. Maalej

**Affiliations:** Psychiatry C Department, Hedi chaker University hospital, sfax, Tunisia

**Keywords:** Psychotic disorders, immigration

## Abstract

**Introduction:**

Many studies showed an increased incidence of psychotic disorders (PD) among immigrants.

**Objectives:**

Study the characteristics of patients suffering from PD and having immigration experience (IE) by comparing them to patients without IE.

**Methods:**

A retrospective controlled study, involving 58 male patients having IE and suffering from PD (DSM 5) who were followed in the psychiatry department of Hedi Chaker University Hospital in Sfax (Tunisia), between January 2013 and December 2018.They were compared to 60 male patients suffering from PD who lack the IE. Samples were matched on age and socio-economic status. Data was collected from their medical records.

**Results:**

The mean age was 36 years old. The countries of destination were almost European (65.5%). The most common PD were schizophrenia (62%) and schizoaffective disorder (19%). The use of psychoactive substances (PS) was noted in 55% of cases.The average time interval between the onset of the PD and immigration was 4,7 years. The mean age of the onset of PD was 27 years old. The mean age of PD onset was earlier among patients having IE (27 year old vs 28.5 year old, p=0.24).The use of PS was significantly higher in these patients (p=0.04).

**Conclusions:**

Our study identified some of the features associated with PD that occur during an immigration experience such as the use of PS. Further studies should be conducted in collaboration with countries of destination of immigrants to clarify the relationship between immigration and PD.

